# Evaluating the Reasoning Capabilities of Large Language Models for Medical Coding and Hospital Readmission Risk Stratification: Zero-Shot Prompting Approach

**DOI:** 10.2196/74142

**Published:** 2025-07-30

**Authors:** Parvati Naliyatthaliyazchayil, Raajitha Muthyala, Judy Wawira Gichoya, Saptarshi Purkayastha

**Affiliations:** 1 Department of Biomedical Engineering and Informatics, Luddy School of Informatics, Computing and Engineering, Indiana University Indianapolis, 535 W Michigan Street, Indianapolis, IN, 46202, United States, 1 317 274 0439; 2 Department of Radiology and Imaging Sciences, Emory University School of Medicine, Emory University, Atlanta, GA, United States

**Keywords:** large language models, clinical decision support, zero-shot learning, medical coding, primary diagnosis prediction, readmission risk prediction, explainability

## Abstract

**Background:**

Large language models (LLMs) such as ChatGPT-4, LLaMA-3.1, Gemini-1.5, DeepSeek-R1, and OpenAI-O3 have shown promising potential in health care, particularly for clinical reasoning and decision support. However, their reliability across critical tasks like diagnosis, medical coding, and risk prediction has received mixed reviews, especially in real-world settings without task-specific training.

**Objective:**

This study aims to evaluate and compare the zero-shot performance of reasoning and nonreasoning LLMs in three essential clinical tasks: (1) primary diagnosis generation, (2) *ICD-9* (*International Classification of Diseases, Ninth Revision*) medical code prediction, and (3) hospital readmission risk stratification. The goal is to assess whether these models can serve as general-purpose clinical decision support tools and to identify gaps in current capabilities.

**Methods:**

Using the Medical Information Mart for Intensive Care-IV dataset, we selected a random cohort of 300 hospital discharge summaries. Prompts were engineered to include structured clinical content from 5 note sections: chief complaints, past medical history, surgical history, laboratories, and imaging. Prompts were standardized and zero-shot, with no model fine-tuning or repetition across runs. All model interactions were conducted through publicly available web user interfaces, without using application programming interfaces, to simulate real-world accessibility for nontechnical users. We incorporated rationale elicitation into prompts to evaluate model transparency, especially in reasoning models. Ground-truth labels were derived from the primary diagnosis documented in clinical notes, structured *ICD-9* codes from diagnosis, and hospital-recorded readmission frequencies for risk stratification. Performance was measured using *F*_1_-scores and correctness percentages, and comparative performance was analyzed statistically.

**Results:**

Among nonreasoning models, LLaMA-3.1 achieved the highest primary diagnosis accuracy (n=255, 85%), followed by ChatGPT-4 (n=254, 84.7%) and Gemini-1.5 (n=237, 79%). For *ICD-9* prediction, correctness dropped significantly across all models: LLaMA-3.1 (n=128, 42.6%), ChatGPT-4 (n=122, 40.6%), and Gemini-1.5 (n=44, 14.6%). Hospital readmission risk prediction showed low performance in nonreasoning models: LLaMA-3.1 (n=124, 41.3%), Gemini-1.5 (n=122, 40.7%), and ChatGPT-4 (n=99, 33%). Among reasoning models, OpenAI-O3 outperformed in diagnosis (n=270, 90%) and *ICD-9* coding (n=136, 45.3%), while DeepSeek-R1 performed slightly better in the readmission risk prediction (n=218, 72.6% vs O3’s n=212, 70.6%). Despite improved explainability, reasoning models generated verbose responses. None of the models met clinical standards across all tasks, and performance in medical coding remained the weakest area across all models.

**Conclusions:**

Current LLMs exhibit moderate success in zero-shot diagnosis and risk prediction but underperform in *ICD-9* code generation, reinforcing findings from prior studies. Reasoning models offer marginally better performance and increased interpretability, with limited reliability. Overall, statistical analysis between the models revealed that OpenAI-O3 outperformed the other models. These results highlight the need for task-specific fine-tuning and need human-in-the-loop checking. Future work will explore fine-tuning, stability through repeated trials, and evaluation on a different subset of deidentified real-world data with a larger sample size.

## Introduction

The rapid evolution of large language models (LLMs), which are artificial intelligence (AI) systems designed to understand and generate human-like text, has sparked widespread interest in their potential applications across various domains [1], particularly health care [2]. Alongside established nonreasoning models like ChatGPT-4, LLaMA-3.1, and Gemini-1.5, new reasoning models, such as DeepSeek-R1 and OpenAI-O3, have also emerged during this study, with reasoning capabilities embedded in their design, enabling more logical, step-by-step decision-making. These models enable users to perform complex language-based tasks without domain-specific training, using only natural language input [3].

While some initial studies highlight the promising ability of these LLMs to handle complex health care tasks [3], others raise critical concerns about their accuracy, reliability, and adherence to the high standards required in clinical settings [4]. This duality highlights the need for careful evaluation of their utility and reliability in real-world clinical environments [5]. This leads us to key questions in this rapidly advancing field: which of these preconfigured LLMs is most suitable for addressing the unique challenges of health care tasks? Do newer reasoning models outperform their nonreasoning counterparts?

To address this question, our study systematically compares the performance of 5 models, prominent nonreasoning LLMs ChatGPT-4, LLaMA-3.1, and Gemini-1.5 as well as reasoning models DeepSeek-R1 and OpenAI-O3 across key health care tasks. The nonreasoning models were selected based on their widespread popularity and adoption, while the reasoning models were chosen for their recently introduced, advanced reasoning capabilities at the time of study design. Specifically, we evaluated their aggregated ability to generate primary diagnoses, code it to the *ICD-9* (*International Classification of Diseases, Ninth Revision*) codes, and predict risk stratification for hospital readmission using zero-shot prompting. To increase interpretability, structured rationale elicitation was incorporated into the prompting for diagnostic and prognostic tasks, especially for nonreasoning models.

In our study context, primary diagnosis refers to the main condition that is chiefly responsible for a patient’s current hospitalization. To ensure consistency across health care systems, diagnosis is coded to *ICD-9* or *ICD-10* (*International Statistical Classification of Diseases, Tenth Revision*) as a standard practice. *ICD-9* and *ICD-10* are standardized coding systems used globally for categorizing diseases, conditions, and medical procedures [6,7]. Each diagnosis is assigned a unique numeric or alphanumeric code that codes diagnoses for medical records. Further, we define hospital readmission as the likelihood of a patient being readmitted to the hospital after discharge within the full time frame covered by the dataset.

This analysis was conducted using the Medical Information Mart for Intensive Care (MIMIC)-IV dataset [8], a controlled-access, real-world clinical dataset derived from critical care hospital admissions. Since these LLMs are primarily trained on publicly available internet data [9], using a controlled-access, real-world, deidentified clinical dataset is better suited for evaluating their clinical performance. This dataset includes detailed patient, admission, diagnosis, and discharge information. From the discharge summaries, also called clinical notes, we extracted sections like chief complaints, past medical history, surgical history, laboratories, and imaging to construct prompts for model evaluation. The primary diagnosis section was excluded from prompts and instead used as ground truth for evaluating diagnostic predictions. Structured *ICD-9* codes served as the reference for code prediction accuracy, while hospital-recorded readmission counts (via hadm_id) were used to assess readmission risk prediction. Zero-shot prompting was used to evaluate model generalizability without task-specific fine-tuning [10,11].

The objective of this study is to evaluate which preconfigured LLMs are most suitable for addressing the unique challenges of health care tasks and whether newer reasoning models outperform their nonreasoning counterparts in predicting primary diagnoses, medical codes, and readmission risk. Additionally, the study aims to assess the potential role of preconfigured LLMs in supporting clinical decision-making without the need for task-specific fine-tuning. By leveraging real-world health care data from the MIMIC-IV dataset and using zero-shot prompting, we evaluate the models’ accuracy and effectiveness in a clinical context. Our analysis seeks to paint a clearer picture of the feasibility and limitations of these models for safer and effective health care applications.

## Methods

### Ethical Considerations

This study involved secondary analysis of deidentified patient data from MIMIC-IV (version 2.2) and MIMIC-IV Note (version 2.2) databases. The Massachusetts Institute of Technology Institutional Review Board approved MIMIC-IV data use (protocol 0403000206). As the dataset is fully deidentified per Health Insurance Portability and Accountability Act requirements, this research was classified as nonhuman participant research, requiring no additional institutional review board approval.

### Study Design

This study used a multistep approach to evaluate the performance of LLMs in addressing key health care tasks, including the ability to predict primary diagnoses, assign *ICD-9* codes, and stratify hospital readmission risks, along with explanations for diagnosis and risk classification. The web-based user interfaces of these LLMs were used, as the study focuses on evaluating readily accessible, out-of-the-box chatbot versions rather than application programming interface (API)–based implementations, which may require additional technical skills and incur extra costs. The methodology is organized into 3 key phases, summarized as follows.

### Sample Collection

**Figure 1.**
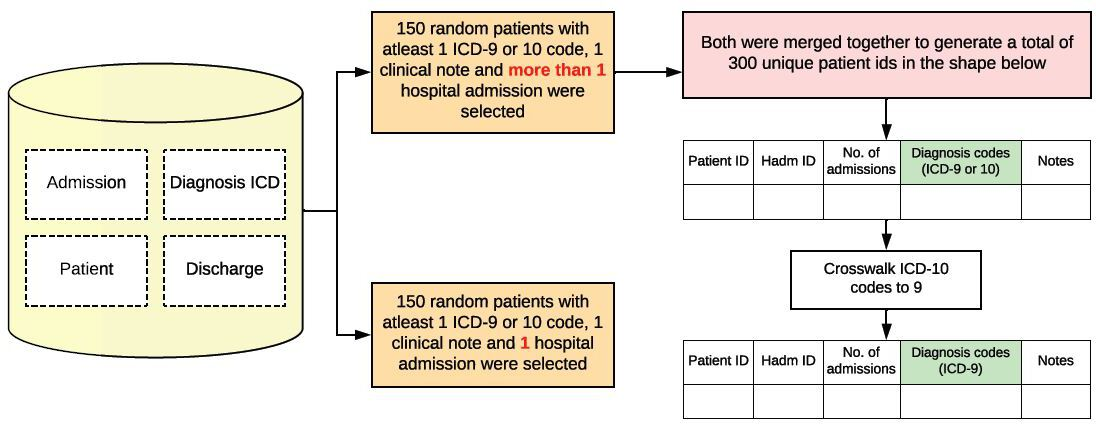
Sample collection of 300 unique subject_ids. This figure shows that the sample of 300 subject_ids was created from the MIMIC-IV dataset, and then, any *ICD-10* (*International Statistical Classification of Diseases, Tenth Revision*) codes in the sample were crosswalked to the respective *ICD-9* (*International Classification of Diseases, Ninth Revision*) using the UMLS crosswalk. The tables show the structure of the output for ease of understanding. MIMIC: Medical Information Mart for Intensive Care; UMLS: Unified Medical Language System.

Clinical data were obtained from the controlled-access MIMIC-IV dataset. It is a deidentified dataset containing detailed health information from patients admitted to the emergency department or intensive care units at Beth Israel Deaconess Medical Center in Boston, MA [12]. A sample of 300 unique patient IDs was selected, ensuring that each patient had valid diagnosis codes and at least 1 available discharge summary. For each patient, *ICD-9* and *ICD-10* codes were extracted as a CSV list, along with their first discharge note. As *ICD-9* codes were more prevalent in the sample, all *ICD-10* codes were crosswalked to *ICD-9* to minimize data loss. Readmission risk was evaluated by calculating each patient’s total number of admissions using hadm_id and admission dates. Of the 300 patients, 150 had multiple admissions, while the remaining 150 had a single admission, as shown in Figure 1. All the subject_ids used in this sample are listed in Multimedia Appendix 1.

### Prompt Template and Creation

**Figure 2.**
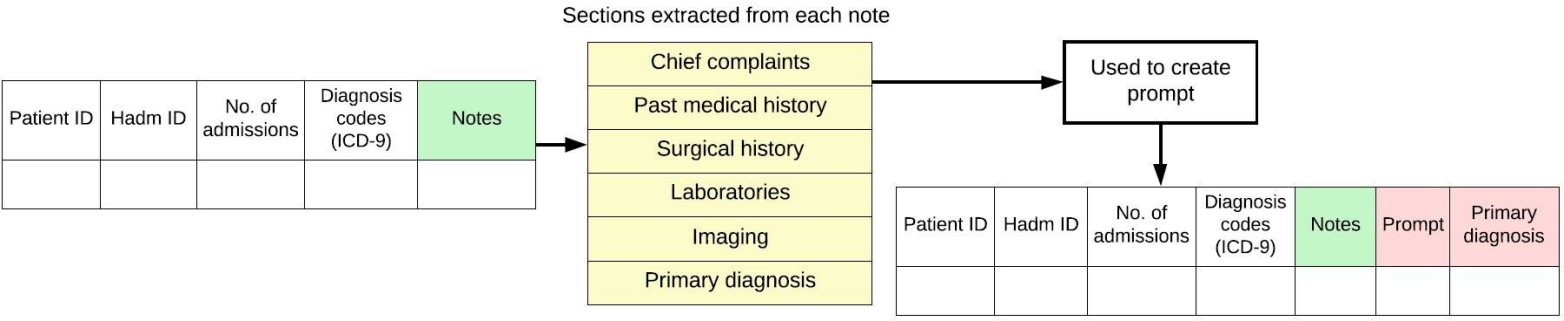
Creation of a prompt using sections from discharge summaries or clinical notes. This figure shows how the output from Figure 1 is further used. The key sections from MIMIC-IV clinical notes were used in prompt creation and extracting the primary diagnosis of the sample. *ICD-9: International Classification of Diseases, Ninth Revision*; MIMIC: Medical Information Mart for Intensive Care.

Prominent sections from labeled discharge summaries in the MIMIC-IV Note database were used to draft prompts [13]. For each patient, the following sections were extracted: chief complaints, past medical history, surgical history, laboratories, and imaging, and programmatically formatted into a structured prompt template for LLM evaluation as shown in Figures2  3. The primary diagnosis was also extracted but not included in the prompt; instead, it served as ground truth for evaluating model performance.

**Figure 3.**
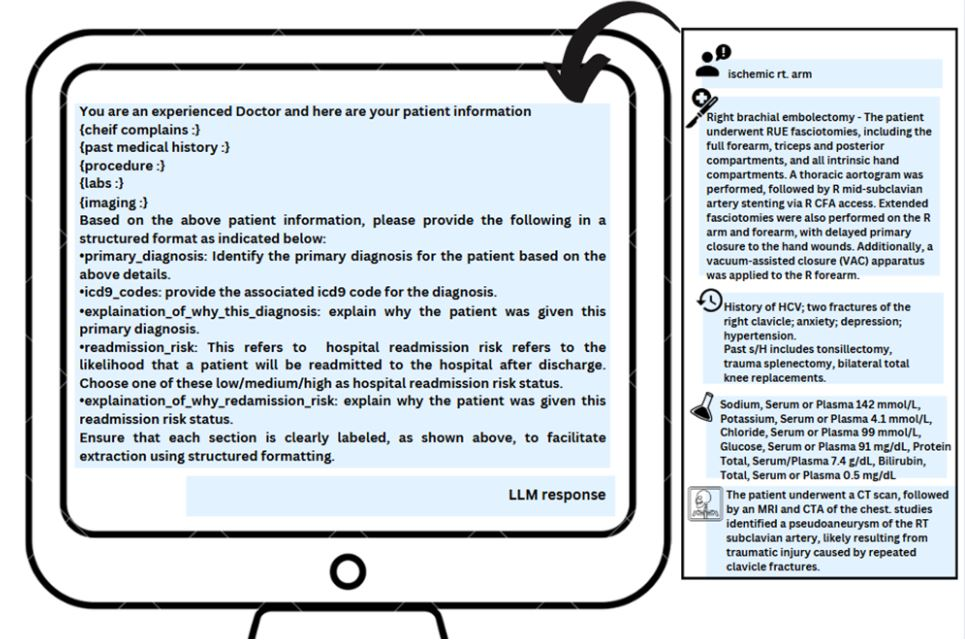
Prompt template. This figure shows the prompt template that is systematically populated for each subject_id from their notes that were extracted. On the right, you see an example representation of MIMIC-IV Note, which is then populated into its respective sections within the prompt. The note here is an example and not an actual record from MIMIC-IV. LLM: large language model; MIMIC: Medical Information Mart for Intensive Care.

To accommodate differences in model context windows, prompt length and content were optimized through preliminary testing. We ensured that essential clinical information was included while keeping the prompt within the context limit among the evaluated models. This balance was critical to maintain fairness across models and to avoid truncation of input. We also tested prompt clarity and effectiveness through pilot runs, refining phrasing and structure to maximize model understanding. Data used in these pilots were excluded from the main research sample. Example prompts are provided in Multimedia Appendix 1.

### Collecting and Processing the Response

All prompts were systematically generated and input into each AI chatbot through their respective web user interfaces. Each prompt was given to each chatbot only once, without repetition, and the memory of the chatbot was disabled to prevent them from learning from each prompt. The generated responses were stored in a CSV file alongside patient metadata. Structured outputs were parsed into individual columns, capturing the primary diagnosis generated by the LLM, a list of *ICD-9* codes associated with the primary diagnosis, the predicted readmission risk status, explanations for the selected primary diagnosis, and justifications for the predicted readmission risk status.

The final dataset was then prepared for evaluation against the ground truth. Detailed prompt structure and response parsing procedures are provided in Multimedia Appendix 1.

## Results

### Overview

This study provides a comparative evaluation of leading LLMs, ChatGPT-4, LLaMA-3.1, Gemini-1.5, DeepSeek-R1, and OpenAI-O3 in terms of their ability to perform health care–specific tasks. The prompt was created from the key sections of MIMIC-IV clinical notes. The responses produced by LLM were extracted into their individual structured columns for analysis and compared against the ground truth from MIMIC-IV data. The results highlight notable and interesting variations in performance across tasks.

### Comparing the Prediction of Primary Diagnosis

The primary diagnosis from each LLM’s response was compared against the primary diagnosis extracted from MIMIC-IV clinical notes. We used SciBERT, a pretrained model specifically designed for scientific and medical contexts [14]. This makes it particularly adept at processing and understanding domain-specific language, which is essential for comparing medical terminologies.

The allenai/scibert_scivocab_uncased variant of SciBERT [14,15], implemented through the SentenceTransformer framework, was used to generate embeddings for both the ground truth primary diagnosis (from MIMIC-IV clinical notes) and the LLM-predicted diagnosis. The process involved:

1. Embedding generation: Both the reference diagnosis and the LLM-generated text were converted into high-dimensional embeddings using SciBERT.
2. Cosine similarity computation: Cosine similarity was calculated between the 2 embeddings to quantify their semantic similarity. A cosine similarity threshold of 0.7 was chosen to reflect a moderate to high level of semantic similarity, ensuring that predictions captured the intended clinical meaning without requiring exact wording. This threshold provided a practical balance between sensitivity and specificity for our evaluation needs. A threshold of 0.7 classifies predictions: scores ≥0.7 were considered semantically aligned with the ground truth and scores <0.7 were categorized as incorrect or divergent predictions.

Among nonreasoning models, LLaMA-3.1 and ChatGPT-4 exhibited comparable performance, with semantic match rates of 85% (255/300) and 84.9% (254/300), respectively. This marginal difference suggests that both models are similarly capable of aligning with the ground truth diagnoses, outperforming Gemini-1.5, which achieved a match rate of 79% (237/300). Between the reasoning models, OpenAI-O3 exhibited higher performance with a 90% (270/300) match rate, whereas DeepSeek-R1 showed an 85% (255/300) match rate. Reasoning models performed better than the nonreasoning models.

### Comparing the Prediction of *ICD-9* Code

To evaluate the accuracy of *ICD-9* code predictions by the LLMs, we performed a systematic comparison against the ground truth codes from the MIMIC-IV dataset, which includes both *ICD-9* codes and *ICD-10* codes. We crosswalked the *ICD-10* codes to *ICD-9* using the Unified Medical Language System [16] *ICD-9* to *ICD-10* crosswalk [7,8]. The decision to crosswalk was driven by the relatively small number of *ICD-10* codes present in our sample, ensuring that the majority of original diagnostic codes could be consistently represented for comparison.

Both the ground truth *ICD-9* codes and LLM-generated codes were converted into CSV lists to ensure uniformity. We then conducted a row-wise comparison to identify matches between the predicted and ground truth *ICD-9* codes.

In evaluating the ability of the nonreasoning LLMs to predict *ICD-9* codes for primary diagnoses, LLaMA-3.1 correctly predicted *ICD-9* codes for 128 of 300 patients. ChatGPT-4 followed, correctly predicting *ICD-9* codes for 122 of 300 patients. Gemini-1.5 lagged behind, predicting *ICD-9* codes for 44 of 300 patients. These results indicate that LLaMA-3.1 and ChatGPT-4 are comparably effective, but their performance still falls short of the accuracy required for reliable medical coding applications, and this finding aligns with studies in the literature [4]. Between the reasoning models, OpenAI-O3 correctly predicted *ICD-9* codes for 136 of 300 patients, whereas DeepSeek-R1 correctly predicted *ICD-9* codes for 121 of 300 patients. The medical coding skills for the reasoning models also lagged far behind the standards expected for clinical practice. Further refinement and training may be needed to enhance the models’ effectiveness in this domain.

### Top 10 *ICD-9* Codes in MIMIC-IV Sample and 3 Nonreasoning LLMs

We evaluated the top 10 *ICD-9* codes from the MIMIC-IV sample and the 3 nonreasoning LLM-generated *ICD-9* codes, as shown in Multimedia Appendix 2. Each subject_id can have multiple *ICD-9* codes. For this analysis, we implemented an *ICD-9* hierarchical rollup by aggregating detailed diagnosis codes to their respective 3-digit parent categories. For example, specific codes like 414.0 (coronary atherosclerosis) and 414.00 (coronary atherosclerosis of unspecified type of vessel) were rolled up to their broader parent category, 414 (other forms of chronic ischemic heart disease). The top 10 *ICD-9* codes were calculated after this rollup.

We found that *ICD-9* codes associated with the parent category 414 (other forms of chronic ischemic heart disease) were present across all 3 LLMs and the MIMIC-IV sample as one of the top 2. In contrast, another parent category, 780 (general symptoms), appeared in all 3 LLMs but was absent in the MIMIC-V sample. This suggests that the LLMs were coding many symptoms differently from clinical practice, highlighting an area for potential improvement. Additionally, the parent category for diabetes mellitus was observed in the MIMIC-IV sample, LLaMA-3.1, and ChatGPT-4, but not in Gemini-1.5, which aligns with our findings of *ICD-9* code predictions, where Gemini-1.5 underperformed.

### Comparing the Prediction of Hospital Readmission Risk Status

The ground truth for readmission risk from MIMIC-IV was derived as a numeric value representing the total number of readmissions per patient. In contrast, the LLM-generated responses were qualitative, assigning each patient a categorical label of low, medium, or high risk. To enable a meaningful comparison between these 2 formats, the numeric readmission counts were converted into qualitative categories. We applied a quantile-based thresholding approach. Specifically, the distribution of readmission counts across the dataset was used to define 3 categories:

- Low risk: Readmission count≤25th percentile
- Medium risk: Readmission count>25th percentile and ≤75th percentile
- High risk: Readmission count>75th percentile

This categorization ensured consistency between the qualitative model outputs and the quantitative ground truth, allowing for structured evaluation of LLM performance in readmission risk prediction.

Among nonreasoning models, LLaMA-3.1 had 41.3% (124/300) correct predictions, followed by Gemini-1.5 with 40.7% (122/300) and ChatGPT-4 with 33% (99/300). While LLaMA-3.1 and Gemini-1.5 demonstrated moderate alignment with the ground truth categories, the overall results suggest significant room for improvement. Among the reasoning models, DeepSeek-R1 performed slightly better with 72.6% (218/300) correct risk predictions than OpenAI-O3 with 70.6% (212/300) correct risk predictions. This shows that reasoning models perform better than nonreasoning models for readmission risk prediction.

### *F*_1_-Score for *ICD-9* Code Prediction and Readmission Risk Status

We calculated the multiclass multilabel *F*_1_-score for *ICD-9* code prediction and the macroaveraged *F*_1_-score for readmission risk stratification for all 5 LLMs. *F*_1_-score for *ICD-9* code prediction helps to evaluate how well the model identifies correct codes while avoiding incorrect ones. For readmission risk prediction, *F*_1_-scores identify how the LLM balances identifying patients at risk (eg, “high risk”) while avoiding unnecessary false alarms. As seen in Table 1, *F*_1_-scores were generally low for both reasoning and nonreasoning models, primarily due to the higher number of false negatives. Among the 3 nonreasoning LLMs, LLaMA-3.1 achieved the highest *F*_1_-scores for both *ICD-9* code prediction and readmission risk stratification. Within the reasoning models, OpenAI-O3 had the highest average *F*_1_-score across both tasks. This finding highlights the fact that, despite some differences in performance, both reasoning and nonreasoning models exhibited notable levels of false negatives and false positives.

**Table 1.** *F*_1_-scores for LLaMA-3.1, ChatGPT-4, and Gemini-1.5^a^.

Chatbot	*F*_1_-score *ICD-9*^b^ code prediction	*F*_1_-score readmission prediction
LLaMA-3.1	0.083	0.412
ChatGPT-4	0.081	0.322
Gemini-1.5	0.024	0.408
DeepSeek-R1	0.091	0.422
OpenAI-O3	0.122	0.414

a This table shows the multiclass multilabel *F*_1_-score for *ICD-9* (*International Classification of Diseases, Ninth Revision*) prediction and *F*_1_-score for hospital readmission risk prediction. *F*_1_-scores take into consideration true positives, true negatives, false positives, and false negatives. The *F*_1_-scores for *ICD-9* code prediction are low for all large language models due to the increased false nega compared to than the true positives.

b *ICD-9*: *International Classification of Diseases, Ninth Revision*.

To evaluate whether the performance differences across models were statistically significant, we initially performed pairwise Wilcoxon signed rank tests on per-task accuracy scores (n=3). After applying Bonferroni correction for multiple comparisons, no pairwise differences reached statistical significance (all corrected *P* values>.05), as shown in Table 2. This lack of significance is likely due to the small number of tasks and limited statistical power. To further assess the robustness of our findings, we also conducted Mann-Whitney *U* tests for independent sample comparisons across all models. The results consistently showed no significant differences between model performances, with *P* values greater than .05 for all pairwise comparisons.

**Table 2.** Pairwise Wilcoxon signed rank test results comparing 5 large language models across 3 tasks^a^.

Model_1	Model_2	Wilcoxon_stat	*P* value	*P* value_bonferroni	Significant
LLaMA-3.1	ChatGPT-4	0.0	.25	2.500000	False
LLaMA-3.1	Gemini-1.5	0.0	.25	2.500000	False
LLaMA-3.1	OpenAI-O3	0.0	.25	2.500000	False
ChatGPT-4	Gemini-1.5	0.0	.25	2.500000	False
ChatGPT-4	OpenAI-O3	0.0	.25	2.500000	False
Gemini-1.5	OpenAI-O3	0.0	.25	2.500000	False
Gemini-1.5	DeepSeek-R1	0.0	.25	2.500000	False
OpenAI-O3	DeepSeek-R1	1.0	.50	5.000000	False
LLaMA-3.1	DeepSeek-R1	1.0	.65	6.547208	False
ChatGPT-4	DeepSeek-R1	2.0	.75	6.547208	False

a This table shows pairwise Wilcoxon signed rank test *P* values and their significance across 3 major tasks.

In addition, to provide a more descriptive analysis of model variability, we computed 95% bootstrap CIs for each model’s mean accuracy. As shown in Table 3, the model OpenAI-O3 achieved the highest average accuracy (69.33%, 95% CI 45.33-90.0), followed by DeepSeek-R1 (65.33%, 95% CI 40.33-85.0). Although LLaMA-3.1 and ChatGPT-4 had lower means (~56%), their CIs overlapped substantially with those of the higher-performing models. Gemini-1.5 demonstrated the lowest performance (42.22%, 95% CI 14.67-79.0), with a wide CI indicating high variability. Together, these analyses suggest that while OpenAI-O3 and DeepSeek-R1 appear to perform better, the limited number of tasks restricts the ability to draw firm conclusions regarding statistical significance. Future studies with a larger and more diverse task set will help validate these trends with greater statistical certainty.

**Table 3.** Bootstrap CIs for each model’s mean accuracy^a^.

Model	Mean accuracy (95% CI)
OpenAI-O3	68.68 (45.33-90.0)
DeepSeek-R1	66.00 (40.33-85.0)
LLaMA-3.1	56.33 (41.33-85.0)
ChatGPT-4	55.41 (40.67-84.9)
Gemini-1.5	42.22 (14.67-79.0)

a This table shows the bootstrap CI for each model’s mean accuracy with OpenAI-O3 showing the top performance when comparing each model toward aggregated tasks.

## Discussion

### Overview

Our results show that reasoning models outperformed nonreasoning ones across most tasks. OpenAI-O3 showed the highest accuracy for primary diagnosis (n=270, 90%) and *ICD-9* coding (n=136, 45.3%), while DeepSeek-R1 led in readmission prediction (n=218, 72.6%). LLaMA-3.1 was the strongest nonreasoning model but showed lower performance on *ICD-9* and readmission tasks. Although statistical significance was not reached, consistent performance trends of reasoning models suggest practical relevance particularly in clinical settings where even small gains can impact outcomes. Reasoning models also provided more detailed explanations, though their verbosity may hinder usability. No model met clinical standards across all tasks. Future work with more tasks and effect size analyses can better validate these patterns.

### Comparison to Prior Work

The existing literature presents mixed findings on the capabilities of LLMs in health care tasks such as diagnosis prediction and medical coding. Soroush et al [4] report poor performance in medical coding, while Kwan [3] showed improved outcomes with augmentation strategies. Lee et al [9] emphasize that while LLMs make errors, they also demonstrate potential in identifying them. Zhu et al [10] illustrate that incorporating longitudinal health records into prompts enhances predictive accuracy. Zhou et al [17] further highlight the value of prompt engineering and fine-tuning with high-quality data for robust diagnostic performance. Nuthakki et al [18] demonstrated that domain-specific deep learning models like Universal Language Model Fine-Tuning, when trained on large-scale datasets such as MIMIC-III, can perform well in ICD code prediction tasks, underscoring the contrast between tailored models and general-purpose LLMs evaluated in our study. Recent studies using MIMIC data also reveal some challenges, one found that converting structured data to free text for mortality prediction with zero-shot prompting showed limited accuracy [19], while another showed that minor changes like word swaps or misspellings can significantly affect ICD code predictions [20]. With these diverse findings in mind, we sought to evaluate the performance of 5 prominent, out-of-the-box LLMs for aggregated high-value health care tasks using a dataset that these LLMs are not already trained on.

Building on prior work, we used a sample size of 300 deidentified patients from MIMIC-IV [21], a larger sample than many previous studies [22]. By leveraging sections of patient discharge summaries and focusing on tasks like predicting primary diagnoses, generating *ICD-9* codes, and stratifying hospital readmission risk, we provide new insights into the potential of LLMs to handle aggregated complex clinical tasks using a chatbot interface, without task-specific fine-tuning. Our use of zero-shot prompting, which avoids the need for additional setup or fine-tuning, highlights the practicality and efficiency of these models in real-world health care settings [23,24]. However, we acknowledge that using publicly available chatbot interfaces rather than controlled APIs or locally hosted models creates challenges for reproducing results. This is because the models behind these tools like ChatGPT-4 are regularly updated and improved without fixed subversion numbers that users can select. Even when accessing the models through APIs, it is not possible to lock in a specific subversion [25], so outputs can change over time. While this limits strict repeatability, it reflects how most real users interact with these models in practice. Our study prioritizes ecological validity over perfect experimental control. For future research, using open-source models like LLaMA-3.1 or DeepSeek-R1 in local environments could help stabilize versions and settings, making experiments easier to reproduce. Our study offers a baseline to understand their strengths, limitations, ethical considerations, and areas for improvement, ultimately guiding future research in fine-tuning and prompt engineering.

### Principal Findings

On evaluating the performance of nonreasoning LLMs for predicting primary diagnoses, LLaMA-3.1 demonstrated improved accuracy, achieving 85% correctness in a zero-shot prompting scenario. While not outstanding, this level of performance demonstrates the model’s capability to support clinical decision-making without task-specific fine-tuning. Between the reasoning models, OpenAI-O3 demonstrated higher performance with 90% correctness. Our approach aims to enhance efficiency and decision-making through AI-human collaboration. Additionally, we generated explanations for each prediction in both reasoning and nonreasoning models to ensure transparency in the model’s reasoning.

DeepSeek-R1 achieved the highest performance in readmission risk prediction (n=218, 72.6%), but the result remains suboptimal, likely in part due to variability within the dataset. Our findings on *ICD-9* prediction align with existing literature [4], which shows that general-purpose LLMs struggle with this task. While OpenAI-O3 (n=136, 45.3%) outperformed other models in *ICD-9* prediction, its low accuracy and modest *F*_1_-score (0.122) highlight the need for improvement, particularly in reducing false positives. This leads us to a central concern with such models, the risk of hallucinations, especially the “faithfulness problem,” where the model generates nonfactual or unfaithful information [26,27]. In high-stakes clinical tasks like medical coding and readmission risk prediction, such hallucinations may lead to misclassification, potentially resulting in suboptimal or even harmful decisions. Automation bias further compounds this risk, as clinicians may overrely on confident but incorrect model outputs without adequate verification [28]. These issues raise important ethical concerns around patient safety, informed oversight, and the responsible deployment of AI in clinical practice. Even minor issues in input, such as word swaps or misspellings in clinical notes, can drastically alter the output [20], especially in the absence of standardized language across clinical documentation. Such vulnerabilities undermine reliability and increase the likelihood of misclassification, particularly in tasks like readmission prediction, where both over- and underestimation can have direct consequences on patient outcomes. Addressing these concerns like miscalculatio,n should be a focus of future research, and we believe that this study offers a valuable foundation. Strategies such as real-time monitoring, feedback loops to flag misclassifications, improved explainability of outputs, and training models on these flagged instances can significantly reduce errors. Incorporating human-in-the-loop or hybrid systems that combine LLMs with clinical expertise may also help prevent misclassifications from escalating. Ultimately, models specifically fine-tuned on clinical text datasets have demonstrated better performance in generating relevant ICD codes and reducing human error, contributing to more accurate documentation, improved patient care, and regulatory compliance [29,30].

Another observation was that reasoning models produced more verbose “explanations” for primary diagnosis and readmission risk than nonreasoning models. Nonreasoning models generated an average of 70 (SD 5.8) words for primary diagnosis explanations and 54 (SD 5.3) words for readmission risk explanations. In contrast, reasoning models like DeepSeek-R1 averaged 418 (SD 56) words for primary diagnosis explanations and 612 (SD 23) words for readmission risk explanations. OpenAI-O3 generated an average of 713 (SD 30) words for primary diagnosis explanations and 1112 (SD 23) words for readmission risk explanations. While transparency and explanation are essential for clinical trust, excessively long responses may increase cognitive load and hinder real-time decision-making, especially for clinicians operating under time constraints. Prior studies have shown that clinicians favor concise, targeted decision support over lengthy narratives, particularly in high-pressure settings [31]. Our findings highlight a trade-off between interpretability and usability [32]. Although we did not include direct feedback from clinicians, future research should incorporate user-centered evaluation metrics such as response usefulness, reading time, and trust perception to better understand how explanation length influences adoption and workflow integration. Tailoring model output length and clarity through prompt design may improve practical adoption and can help strike a balance between clarity and efficiency.

### Strengths and Limitations

Our study used a deidentified dataset to protect patient privacy and confidentiality. However, from an ethical and operational perspective, deploying LLMs in real health care systems raises pressing questions. These include how to protect patient privacy, ensure informed consent, and avoid automation bias or overreliance on potentially hallucinated or unvalidated outputs. Automation bias can lead clinicians to accept AI-generated suggestions without sufficient scrutiny, particularly concerning when LLMs hallucinate plausible-sounding but incorrect diagnoses or codes [28]. Recent findings also show that LLMs like GPT-4 fail to adequately represent demographic diversity in clinical scenarios, often reinforcing stereotypes in race- and gender-based presentations of disease [33]. A related phenomenon, “shortcut learning,” where AI models may rely on spurious features rather than true clinical signals, further complicates these issues, generating biased outcomes even when protected attributes are not explicitly used as inputs [34]. Shortcut learning introduces various biases across different phases of AI development, including data bias, modeling bias, and inference bias [34]. Resolving these ethical challenges requires a multifaceted approach: establishing transparent model auditing processes, enforcing rigorous data governance policies, clinician-in-the-loop frameworks, and ensuring that patients and clinicians are adequately informed about the use and limitations of AI tools. Effective deployment of fairness assessments requires comprehensive bias audits across demographic subgroups, transparent model evaluation, and active mitigation strategies by deploying bias mitigation tools [34,35]. Emerging legal frameworks emphasize accountability for biased AI models, underscoring the necessity for comprehensive fairness assessments [34]. Interdisciplinary collaboration among ethicists, clinicians, and AI developers will be essential to ensure that these tools are not only technically effective but also fair, trustworthy, and aligned with clinical standards [35,36]. Future work should focus on further evaluating these biases by leveraging emerging bias detection tools, refining existing mitigation strategies, and developing accessible, domain-specific frameworks tailored for clinical use.

Additionally, models like Gemini-1.5 showed a safety-first behavior with the response of “Call or text 988 for support” when prompted with scenarios involving psychiatric information. While ethically commendable, this may limit utility in some care contexts. This reveals a deeper tension between safety safeguards and task performance that future models must navigate. The challenge lies in balancing the model’s need to err on the side of caution to avoid harm while ensuring it provides relevant and actionable insights for health care professionals. Addressing this issue can perhaps be done through the development of more context-sensitive responses or clinician-in-the-loop models that can help mitigate this tradeoff.

### Conclusions

This study provides a nuanced understanding of the strengths and limitations of LLMs in health care tasks using zero-shot diagnostic prompting. While none of the models met clinical performance thresholds out of the box, their varied capabilities, particularly LLaMA-3.1’s consistent performance among nonreasoning models and OpenAI-O3’s strength across reasoning tasks, underscore the potential for leveraging LLMs in clinical workflows with minimal setup. However, the reliance on the MIMIC-IV dataset, which reflects a single-center and deidentified hospital population, may limit the generalizability of these findings to broader or more diverse health care settings.

These results reinforce the need for further adaptation of LLMs through domain-specific training, enhanced data preprocessing (eg, standardizing clinical note structures), and fine-tuning with clinical datasets to improve contextual understanding and minimize hallucinations. Incorporating a real-time flagging system and clinician-in-the-loop frameworks could also enhance safety, usability, and trust. Future work will focus on refining models for hospital readmission risk prediction, evaluating their reasoning quality, and exploring hybrid systems that combine LLM outputs with expert oversight to better align with clinical standards and support reliable decision-making. The limitations identified in this study serve as critical guideposts for shaping future research, ultimately moving the field closer to the safe and effective clinical integration of LLMs.

## Supplementary material

10.2196/74142 Multimedia Appendix 1 Additional information.

10.2196/74142 Multimedia Appendix 2 Nonreasoning large language models (LLMs) and Medical Information Mart for Intensive Care (MIMIC)-IV sample top 10 *ICD-9* (*International Classification of Diseases, Ninth Revision*) codes. This figure shows the top 10 *ICD-9* codes from MIMIC-IV sample and the 3 nonreasoning LLMs. Such graphs can help us show patterns. Here, we see a pattern of ischemic heart diseases showing in the MIMIC-IV sample and LLM, whereas the category of general symptoms was only seen in all 3 LLMs and not MIMIC-IV, showing that it might be an area for scope of improvement. Diabetes mellitus is seen in MIMIC-IV sample, LLaMA-3.1, and ChatGPT-4 and not in Gemini-1.5, which aligns with our findings of *ICD-9* code predictions where Gemini-1.5 underperformed.

## Abbreviations

AI: artificial intelligence
API: application programming interface
*ICD-10*: *International Classification of Diseases, Tenth Revision*
*ICD-9*: *International Classification of Diseases, Ninth Revision*
LLM: large language model
MIMIC: Medical Information Mart for Intensive Care
