# Supplementary material for: Evaluating the Reasoning Capabilities of Large Language Models for Medical Coding and Hospital Readmission Risk Stratification: Zero-Shot Prompting Approach
Source: J Med Internet Res. 2025 Jul 30;27:e74142. doi: 10.2196/74142 (PMC12310144; doi:10.2196/74142)
Supplement: Multimedia Appendix 1 [file jmir-v27-e74142-s001.docx]

**MIMIC-IV subject_ids used in this study are listed below to help reproducibility of findings.**

10257709,10337761,10429673,10448948,10577827,10653374,10682684,10702026,10762397,10862054,10866696,10918906,11031754,11069015,11072213,11084430,11131101,11163466,11189676,11214311,11276540,11280077,11451795,11478069,11487835,11532808,11553072,11594102,11618548,11625955,11632582,11889439,11953698,11956536,11978716,12118241,12151073,12323168,12369221,12399776,12487389,12491172,12557139,12670269,12790721,12801290,12819232,12881658,12991500,13221453,13245918,13483571,13485127,13568094,13570759,13592605,13740018,13774492,13805528,13814530,13956197,14045846,14138854,14213287,14375008,14420647,14453342,14476900,14532157,14632617,14689761,14720255,14737153,14847272,14857511,14876226,14884535,14910490,14936002,15006102,15114092,15120026,15153615,15186614,15192197,15213209,15278197,15360405,15396101,15396651,15427916,15496215,15527705,15554479,15834912,15862403,15916814,15937220,15996479,16087867,16121672,16131218,16191519,16237480,16302564,16388704,16512720,16577742,16731679,16738437,16758446,16876939,16879269,16988043,17021029,17221167,17239322,17273122,17356651,17424221,17464265,17623748,17643850,17762007,17854623,17866685,17869704,17934440,17960640,18082575,18167363,18188204,18253639,18350105,18415840,18428011,18564645,18591383,18649959,18697040,18729731,18896861,19162571,19199309,19236898,19299142,19410285,19502402,19544020,19599279,10041135,10041407,10045574,10073680,10083435,10140771,10261340,10368757,10450072,10460043,10487877,10674201,10685798,10738109,10748293,10769428,10789600,10803827,10905492,10984109,11144523,11180120,11345974,11402807,11541214,11746942,11787231,11859746,11977174,12094244,12168621,12213330,12234376,12255815,12354812,12420094,12423675,12488534,12705081,12740060,12801896,12939877,12971125,13075457,13080214,13082004,13146486,13235045,13239528,13268354,13296785,13313653,13397409,13560750,13734102,13749092,13800998,13916620,13954265,14124670,14148873,14203967,14278128,14308157,14341337,14381388,14414041,14460701,14534236,14545284,14571459,14586446,14608217,14624317,14812282,14984178,15127746,15202789,15251571,15286662,15372856,15416363,15487360,15547384,15561083,15806489,15844577,15898052,15912409,15920069,16004662,16189796,16221749,16226050,16283741,16336618,16345832,16439868,16482715,16586311,16586451,16706232,16744866,16746061,16824365,16843842,17042033,17078988,17130064,17300746,17481223,17531008,17798298,17904156,17963237,17993271,18089045,18121486,18121608,18159815,18161155,18167441,18339151,18365879,18458009,18561697,18671922,18683315,18713323,18757698,18862588,18959535,18968832,19048392,19216383,19234360,19257824,19374186,19374927,19458383,19466576,19562059,19599211,19665058,19712371,19738738,19757967,19956599,19962726,19985513

**ICD-9- ICD-10 Crosswalk from UMLS that is used to crosswalk a few ICD-10 codes in MIMIC-IV diagnosis to ICD-9.**

The MIMIC-IV sample of 300 subject_ids had a combination of ICD-9 and ICD-10 codes. We choose to crosswalk ICD-10 codes to ICD-9 to have only one terminology. We choose to stick with ICD-9 codes as those were the majority codes in our sample. To reduce the variation created due to crosswalks and keep as many as original codes for analysis, we choose to use ICD-9 codes. This crosswalk is extracted from UMLS. Due to its larger file size, it is attached as a separate file with submission.

**MIMIC-IV Data structure and Prompt creation details**

MIMIC-IV is a deidentified dataset containing patient records from intensive care units. It includes structured clinical data such as demographics, diagnoses, procedures, laboratory test results, etc., as well as unstructured data like labeled clinical notes[12]. Key files related to admissions, patient, diagnoses_icd, and discharge were utilized in conjunction to create our sample. A sample of 300 subjets were created, ensuring each patient had valid diagnosis codes and at least one available discharge summary. All ICD-10 codes were crosswalked to ICD-9. To evaluate hospital readmission risk, each patient's total number of admissions is calculated using *hadm_id*, and admission dates. In the 300 patient sample, 150 patients had more than one readmission, while the remaining 150 did not experience any readmission.

MIMIC-IV Notes provides preprocessed and labeled clinical notes, offering a rich source of information about a patient's clinical journey[13]. For each patient, the following sections were extracted: chief complaints, past medical history, surgical history, labs, and imaging. Since these sections were labeled, we used custom regex to extract these sections. These extracted sections, except for primary diagnosis, were then programmatically integrated into a structured "prompt template" for large language model (LLM) evaluation. All the responses from the LLM were recorded into CSV to be validated against the ground truth.

**Prompt Structure:**

You are an experienced Doctor and here is your Patient Information

Chief complaints:{}

Past medical hisory: {}

Procedure: {}

Labs: {}

Imaging: {}

Based on the above patient information, please provide the following in a structured format as indicated below:

primary_diagnosis: identify the primary diagnosis for the patient based on the provided sections. icd9_codes: associated with the diagnosis.

explaination_of_why_this_diagnosis: explain why you think.

readmission_risk: choose one of these low/medium/high. explaination_of_why_redamission_risk: explain why you think.

Ensure that each section is clearly labeled, as shown above, to facilitate extraction using structured formatting. Do not provide any further explanation beyond what’s asked.

**Examples of Prompt with Chatbot responses**

Example 1:

**Prompt :** You are an experienced Doctor and here is your Patient Information

Chief Complaints: SOB; cough.

Past Medical History: None

Procedure: Transbronchial biopsy of lung mass.

Labs: 09:45AM BLOOD WBC-9.1 RBC-5.12 Hgb-15.1 Hct-45.5 MCV-89 MCH-29.6 MCHC-34.3 RDW-14.1 Plt Neuts-63.2 Monos-3.7 Eos-5.7* Baso-0.8 Glucose-176* UreaN-19 Creat-0.9 Na-137 K-5.9 Cl-102 HCO3-23 AnGap-17, BLOOD Albumin-4.3 Calcium-9.7 Phos-4.7* Mg-2.1, BLOOD ALT-19 AST-40 LD(LDH)-421* AlkPhos-73 TotBili-0.3, BLOOD Lactate-3.3*.

Imaging: CT Chest w/o con: 1. Large; infiltrative right hilar mass; probably bronchogenic carcinoma; encasing the entire right bronchial tree; spreading into the adjacent right upper lobes; metastasized to conglomerate right upper and lower paratracheal conglomerate lymphadenopathy threatening the superior vena cava and the contralateral prevascular mediastinal nodes. Given the rapid growth of mediastinal nodes on conventional radiographs this is a very aggressive tumor; probably poorly differentiated. 2. Small left pulmonary nodules and left adrenal nodule; are likely metastases. No bone lesions. 3. Right lower lobe tumor infiltration.

Based on the above patient information, please provide the following in a structured format as indicated below:

primary_diagnosis: identify the primary diagnosis for the patient based on the provided sections. icd9_codes: associated with the diagnosis.

explaination_of_why_this_diagnosis: explain why you think.

readmission_risk: choose one of these low/medium/high. explaination_of_why_redamission_risk: explain why you think.

Ensure that each section is clearly labeled, as shown above, to facilitate extraction using structured formatting. Donot provide any further explaination beyond thats asked.

**LLaMA-3.1 Response:**

primary_diagnosis: Bronchogenic carcinoma (lung cancer) with metastasis

icd9_codes:

162.3 (Malignant neoplasm of lung, unspecified),

196.0 (Secondary malignant neoplasm of mediastinum),

196.8 (Secondary malignant neoplasm of other specified sites)

explaination_of_why_this_diagnosis: The patient's CT Chest scan reveals a large, infiltrative right hilar mass, likely bronchogenic carcinoma, with metastasis to lymphadenopathy and other sites (left pulmonary nodules, left adrenal nodule). Labs indicate elevated WBC and lactate levels, suggesting infection or inflammation. The aggressive nature of the tumor and rapid growth indicate poorly differentiated cancer.

readmission_risk: High

explaination_of_why_readmission_risk: Aggressive nature of the tumor

Presence of metastasis

Potential complications (obstructive pneumonia, hemorrhage)

Elevated lactate levels

Rapid growth of mediastinal nodes.

Example 2:

**Prompt:** You are an experienced Doctor and here is your Patient Information

Chief Complaints: Pancreatic mass.

Past Medical History: Diabetes dx ; Stress incontinence; Hyperlipidemia.

Past Surgical History: LLE VNUS; bladder sling; Vitrectomy; D&C; Cataract removal.

Procedure: EGD with duodenal stent insertion.

Labs: 07:40PM BLOOD WBC-12.6* RBC-4.87 Hgb-14.5 Hct-47.0 MCV-92 MCH-27.7 MCHC-32.2 RDW-13.3 Plt Glucose-143* UreaN-8 Creat-0.6 Na-139 K-4.6 Cl-107 HCO3-13* AnGap-24*, BLOOD ALT-21 AST-19 AlkPhos-83 Amylase-56 TotBili-0.3, BLOOD Lipase-132* , BLOOD Calcium-9.8 Phos-3.4 Mg-1.6 , BLOOD CEA-35*

Imaging: EDG Impression: Normal mucosa in the esophagus and stomach There was erythematous mucosa in D2 with duodenal narrowing consistent with a malignant stricture. A 12 mm balloon was passed beyond this point and inflated. Contrast was injected and confirmed a D2 stricture. A 95 mm by 21 mm metal uncovered duodenal stent (Ref 6502; was placed successfully under fluoroscopic and endoscopic guidance. Fluoroscopy confirmed excellent placement. A waist was seen. Otherwise normal EGD to third part of the duodenum. CYTOLOGY: Pending Imaging:

Based on the above patient information, please provide the following in a structured format as indicated below:

primary_diagnosis: Identify the primary diagnosis for the patient based on the provided sections. icd9_codes: associated with the diagnosis.

explaination_of_why_this_diagnosis: explain why you think.

readmission_risk: choose one of these low/medium/high. explaination_of_why_redamission_risk: explain why you think.

Ensure that each section is clearly labeled, as shown above, to facilitate extraction using structured formatting. Donot provide any further explaination beyond thats asked.

**ChatGPT-4 Response:**

primary_diagnosis:

Pancreatic mass with duodenal stricture

icd9_codes:

- 157.9 - Malignant neoplasm of pancreas, unspecified

- 569.3 - Stricture of duodenum

- 250.00 - Diabetes mellitus without complications

- 275.9 - Disorder of lipid metabolism, unspecified

explanation_of_why_this_diagnosis:

The primary diagnosis is a pancreatic mass causing a duodenal stricture, as evidenced by the findings of erythematous mucosa and narrowing in the second portion of the duodenum (D2) during EGD. The placement of a duodenal stent confirms the presence of a malignant stricture. Additionally, the patient’s elevated CEA and lipase levels support the possibility of a pancreatic malignancy affecting the gastrointestinal tract.

readmission_risk:

High

explanation_of_why_readamission_risk:

The patient’s pancreatic mass, along with the duodenal stricture, suggests the presence of a malignancy, which carries a high risk for complications such as further obstruction, infection, or metastatic spread. The elevated CEA and lipase levels also suggest ongoing or progressive disease. Given the need for ongoing management and possible recurrence or complications, the readmission risk is considered high.

**Additional Figures**

Figure S1: Comparative analysis of Accuracy of Primary Diagnosis Prediction


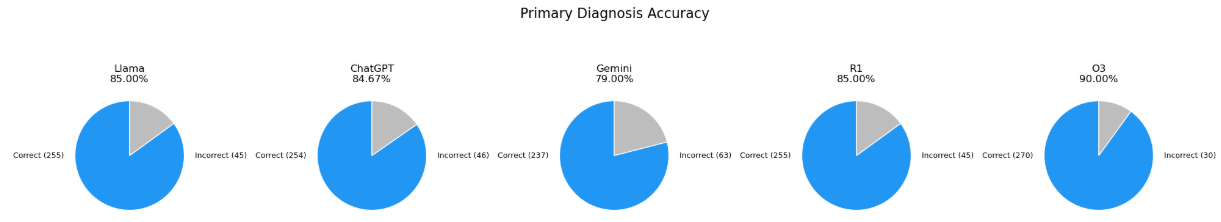


Figure S2: Comparative analysis of Accuracy of ICD-9 Code Prediction


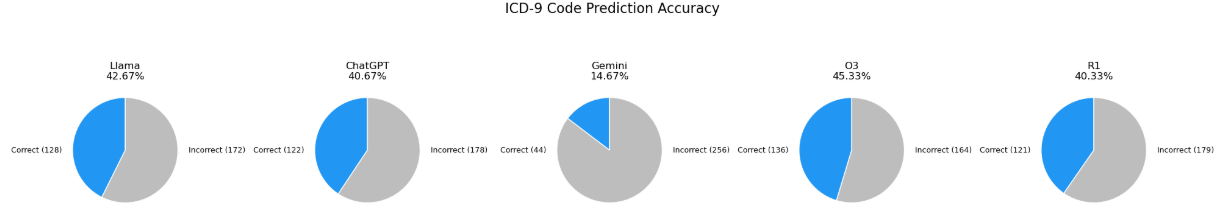


Figure S3: Comparative analysis of Accuracy of Readmission Risk Prediction


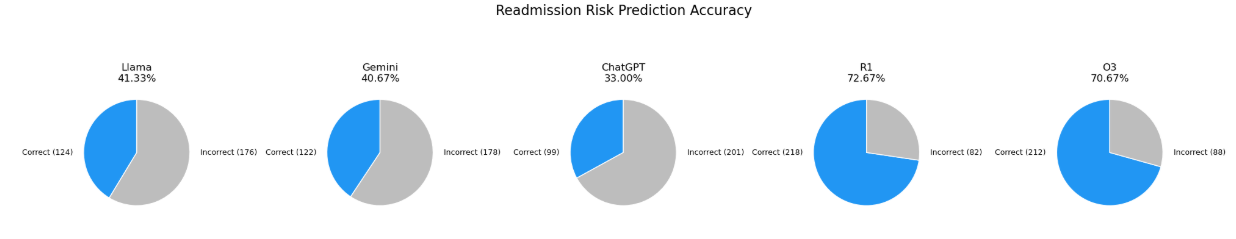


**Additional Tables**

Table S1: Pairwise Mann–Whitney U test results comparing five LLMs accuracies across three task

| Model_1 | Model_2 | U_stat | p_value | p_value_bonferroni | Significant |
| --- | --- | --- | --- | --- | --- |
| LLaMA-3.1 | ChatGPT-4 | 7.0 | 0.375825 | 3.758251 | False |
| ChatGPT-4 | Gemini-1.5 | 7.0 | 0.375825 | 3.758251 | False |
| ChatGPT-4 | OpenAI-O3 | 2.0 | 0.375825 | 3.758251 | False |
| LLaMA-3.1 | Gemini-1.5 | 7.0 | 0.400000 | 4.000000 | False |
| LLaMA-3.1 | OpenAI-O3 | 2.0 | 0.400000 | 4.000000 | False |
| Gemini-1.5 | OpenAI-O3 | 2.0 | 0.400000 | 4.000000 | False |
| Gemini-1.5 | DeepSeek-R1 | 2.0 | 0.400000 | 4.000000 | False |
| OpenAI-O3 | DeepSeek-R1 | 5.0 | 1.000000 | 10.000000 | False |
| LLaMA-3.1 | DeepSeek-R1 | 4.5 | 1.000000 | 10.000000 | False |
| ChatGPT-4 | DeepSeek-R1 | 4.0 | 1.000000 | 10.000000 | False |

^This table shows each model's performance was evaluated on three tasks (n = 3). P-values represent significance of differences in accuracy distributions.^

Table S2: Consolidated Table of all Results for all models evaluated

| Model | Accuracy diagnosis | Accuracy ICD9 code | Accuracy readmission risk | F1 score ICD9 | F1 score readmission risk |
| --- | --- | --- | --- | --- | --- |
| LLaMA-3.1 | 85% | 42.7% | 41.3% | 0.083 | 0.412 |
| ChatGPT-4 | 84.9% | 40.7% | 40.6% | 0.081 | 0.322 |
| Gemini-1.5 | 79% | 14.6% | 33% | 0.024 | 0.408 |
| OpenAI-O3 | 90% | 45.3% | 70.7% | 0.091 | 0.422 |
| DeepSeek-R1 | 85% | 40.3% | 72.67 | 0.122 | 0.414 |

^The table reports per-task accuracy scores for each model across the three evaluation tasks along with their F1 Scores^
