# Supplementary figures and images for: Evaluating the Reasoning Capabilities of Large Language Models for Medical Coding and Hospital Readmission Risk Stratification: Zero-Shot Prompting Approach
Source: J Med Internet Res. 2025 Jul 30;27:e74142. doi: 10.2196/74142 (PMC12310144; doi:10.2196/74142)

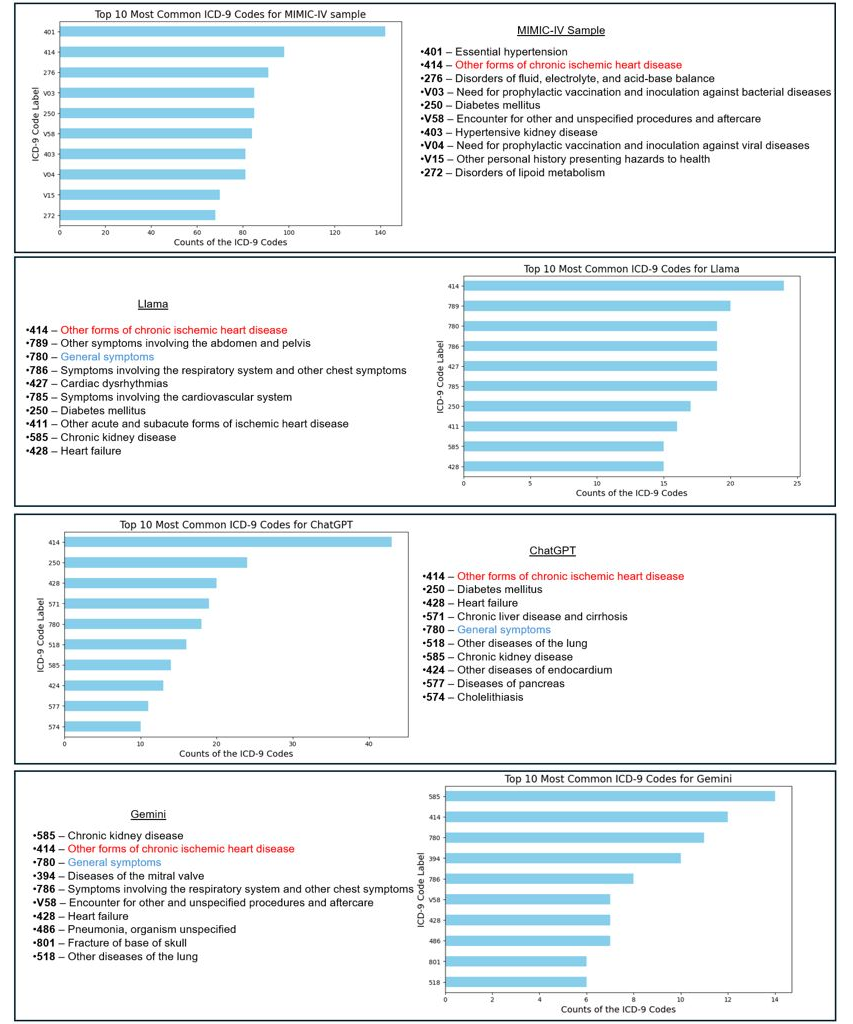

Supplement: Multimedia Appendix 2 [file jmir-v27-e74142-s002.png]
